# The reciprocal relationship between mathematics self-efficacy and mathematics performance in US high school students: Instrumental variables estimates and gender differences

**DOI:** 10.3389/fpsyg.2022.941253

**Published:** 2022-09-22

**Authors:** Chris Sakellariou

**Affiliations:** Nanyang Technological University, Singapore, Singapore

**Keywords:** mathematics achievement, mathematics self-efficacy, longitudinal data, unobserved heterogeneity, gender differences

## Abstract

**Objective:**

To investigate the reciprocal relationship between high school students’ academic self-efficacy and achievement in mathematics using US data from the HSLS:2009 and first follow-up longitudinal surveys, while accounting for biases in effect estimates due to unobserved heterogeneity.

**Methods:**

Instrumental Variables (IV) regressions were estimated, to derive causal effect estimates of earlier math self-efficacy on later math achievement and vice versa. Particular attention was paid to testing the validity of instruments used. Models were estimated separately by gender, to uncover gender differences in effects.

**Results:**

Evidence of robust reciprocal effects between self-efficacy and achievement for male students is presented, with the dominant effect from earlier achievement to later self-efficacy. For girls, evidence of such effects is weak. Generally, IV estimates are higher than OLS estimates for males, but not for females. As opposed to earlier correlational studies which did not find significant gender differences despite theoretical expectations for their existence, the findings support higher effects for male students.

## Introduction

The theoretical and empirical literature has examined the role of non-cognitive, domain-specific constructs, referred to as student self-beliefs (such as self-efficacy and self-concept), in predicting behavior, choices, and practices which can affect achievement. Bandura (1982) defines perceived general self-efficacy as a personal judgment of “how well one can execute courses of action required to deal with prospective situations.” Self-efficacy is domain-specific and multidimensional, and beliefs vary across wide ranges of activities. Academic self-efficacy, as opposed to general self-efficacy, relates to students’ confidence in their ability to complete academic tasks like studying for examinations and writing term papers, and should be measured in academic settings (e.g., Animasaun and Abegunrin, 2018). Developmental origins of self-efficacy perceptions include familial sources, peer influences, and transitional influences of adolescence. Since academic self-efficacy influences students’ approach to confronting educational challenges, a higher academic achievement is expected for students with higher self-efficacy (Pajares and Schunk, 2001). Past studies have described self-efficacy as a positive predictor of performance outcomes (e.g., Schunk et al., 2008). According to Usher and Pajares (2008), self-efficacy “predicts students’ academic achievement across academic areas and levels. Past successes in (non-trivial) tasks consistent with mastery in a particular domain (e.g., mathematics) increase students’ confidence in succeeding again. In other words, self-efficacy is likely both the cause and the effect of academic achievement (see Pajares and Schunk, 2002). Student motivation is interlinked with specific student self-efficacy beliefs (for example, in doing well in a mathematics test) and such beliefs do not coincide with students’ academic ability, since they are self-constructed.

It has been proposed that there may be too much or too little self-efficacy and that the optimum level seems to be slightly above a student’s true capacity (Bandura, 1977). Students may and often do misperceive their true skills, which could have complex effects on students’ motivations (Pajares, 1996, p. 565). Grossly overestimating self-efficacy might lead a student not preparing for a task properly, which will impair performance. Emerging evidence suggests that men overestimate both their ability as well as their perception of past performance, while women either underestimate both or at least rate them more accurately, while in most situations, actual performance in mathematics does not differ significantly between genders (e.g., Bench et al., 2015). Chatard et al. (2007) found that, the more students believed in gender stereotypes prior to recalling their marks, the more female students underestimated their marks in mathematics and male students underestimated their marks in arts. With respect to empirical evidence on how biases in self-evaluations of math competence relate to achievement, there is no consensus. Advocates of the view that being optimistic about one’s efficacy is required for a variety of accomplishments and is associated with higher motivation, persistence and performance include Taylor and Brown (1988) and Bandura (1993). The opposite view states that overly optimistic or overly pessimistic self-assessments may lead disappointment and loss of morale, following repeated failures (Schunk, 1981). Related empirical findings are mixed. Some studies found that overstating one’s competence is associated with higher academic performance (e.g., Martin and Debus, 1998). On the other hand, Robins and Beer (2001) found no association between overstating competence and receiving better grades or likelihood of graduation. Dupeyrat et al. (2011) found that overrating one’s mathematics competence was related to better mathematics achievement, although the findings were open to interpretation.

Section “Self-efficacy and mathematics achievement using longitudinal data” reviews the more recent literature investigating reciprocal effects between self-efficacy and achievement using longitudinal—repeated measure data. Section “Contribution of study and objectives” outlines the contribution and objectives of the study.

### Self-efficacy and mathematics achievement using longitudinal data

Studies on the relationship between self-beliefs and performance often refer to the notion of reciprocal determinism at a theoretical level (Bandura, 1986). However, the reciprocal relationship between self-efficacy and performance has not found direct empirical support; this could be due to the relative scarcity of longitudinal, repeated-measure data, as opposed to cross-sectional data. For example, Williams and Williams (2010) investigated the reciprocal determinism of self-efficacy and mathematics achievement in 15-year-old students using cross-sectional data.

Of the models which assume a causal relationship between self-efficacy and achievement, the *self-enhancement model* proposes that the dominant effect is through high prior self-efficacy enhancing achievement in a particular domain. The *skill development model*, on the other hand, proposes that there is a causal association between achievement and future self-efficacy, because academic success (failure) improves (diminishes) self-efficacy (Usher and Pajares, 2008). The *reciprocal-effects model* stresses the likely role of self-efficacy as both a cause of and an effect of academic achievement and intends to integrate the causal relationships proposed by the two alternative models (Marsh, 1990).

Various methodological approaches have been used in assessing the relationship between self-efficacy and achievement, including meta-analytic studies, studies using longitudinal-repeated measures data and approaches such as structural equation modeling. Valentine et al. (2004) synthesized the findings of longitudinal studies investigating the relationship between self-beliefs and achievement and found a small influence of generalized positive self-beliefs on academic achievement after controlling for initial levels of achievement, and somewhat stronger effects when measures of self-beliefs and achievement are matched by academic domain. Valentine and DuBois (2005) found stronger evidence for the *skill development model* compared to the *self-enhancement model*.

More recent studies using longitudinal data include Hannula et al. (2014), Hwang et al. (2016), and Schöber et al. (2018). Hannula et al. (2014) used Finish longitudinal data and autoregressive/cross-lagged models to estimate the reciprocal causal relationship between mathematics enjoyment, mathematics self-efficacy, and mathematics achievement. They found that mathematics achievement and self-efficacy have a reciprocal relation and that the dominant effect is from achievement to self-efficacy. Hwang et al. (2016) used data on Korean students in the liberal arts track and an autoregressive cross-lagged model to assess the casual ordering of self-efficacy beliefs and academic achievement over short time intervals. Performance scores were from Korean, English, mathematics, and social studies subjects. They found a reciprocal relationship, with the effect of past academic achievement on self-efficacy beliefs being stronger than the effect of self-efficacy beliefs on academic achievement. One of the reported limitations of the study is that the design of the study does not allow for a clear conclusion regarding the reciprocal causal influence between self-efficacy and academic achievement, as it does not account for unobserved confounding factors. Schöber et al. (2018), did not find support for the *skill development model*, while their estimate of the positive effect of mathematics self-efficacy on later mathematics achievement was small and in line with that of Valentine et al. (2004). The authors state that, since the influence of self-efficacy on achievement is mediated by other variables and such variables were not available, the processes underlying the causal link from self-efficacy to later achievement and vice versa could not be examined. Furthermore, the findings of the study (lack of consistent reciprocal effects) may be due to the short interval between measurement occasions.

One recent study by Zakariya (2021) aimed to derive evidence of a causal relationship between self-efficacy and performance in mathematics, using an instrumental variable approach to structural equation modeling. Unfortunately, besides the small sample size, the findings (large bidirectional effects between math self-efficacy and math performance) are not generalizable, as the focus is on first-year engineering students following a mathematics course at a Norwegian university. Finally, the above cited studies were not designed to derive gender-specific evidence on reciprocal effects.

### Contribution of study and objectives

Reflecting on the empirical literature, estimation of the reciprocal causal relationship between self-efficacy and achievement benefits from the use of longitudinal data with multiple measurement occasions and adequate time interval between measurements. Furthermore, the methodology, needs to account for the potential endogeneity of students’ self-efficacy perceptions and mathematics performance, when attempting to estimate bidirectional causal effects between mathematics self-efficacy perceptions and mathematics achievement. By endogeneity, I refer to a covariate appearing in a model, which is correlated with unobserved student characteristics which also affect the outcome. While the terms endogeneity and unobserved heterogeneity often used interchangeably, unobserved heterogeneity refers to variation/differences among subject participants which are unmeasured, hence omitted from the model.^1^ Endogeneity, on the other hand, can arise from three sources^2^: omitted variables, measurement error, and simultaneity. Presence of any of the three, or a combination of them can bias effect estimates. Hence one needs to use an estimation method which accounts for potential endogeneity of covariates of interest, to derive bias-corrected effect estimates. In this investigation, mathematics self-efficacy perceptions (when assessing the effect of earlier self-efficacy on later performance) and achievement (when assessing the effect of earlier performance on later self-efficacy) are treated as potentially endogenous covariates.

The following research questions were investigated:

**RQ1**: Is there evidence that students’ self-efficacy perceptions (Model 1) and/or students’ mathematics achievement (Model 2) are endogenous covariates? If this is the case, the OLS effect estimates will be biased, and one needs to use an estimation method which accounts for such biases.

***RQ2***: Is there evidence in support of a reciprocal relationship between academic self-efficacy and academic achievement?

***RQ3***: Are there gender differences in relation to RQ1 and RQ2?

## Materials and methods

Section “Data: The HSLS:09 and first follow-up” provides information on the HSLS:09 and follow up surveys and associated datasets, while Section “Methodological approach” outlines the methodological approach.

### Data: The HSLS:09 and first follow-up

I used data from the US High School Longitudinal Study (HSLS:09) and the 2012 follow up, in which students who were in grade 9 during the 2009–2010 school year were followed for 7 years into high school graduation, and college or employment. The survey, implemented by the U.S. National Center for Education Statistics (NCES) contains measures of students’ mathematics performance (mathematics assessment in algebraic reasoning) and mathematics self-efficacy beliefs, both at two points in time (at grades 9 and 11), which is needed for testing reciprocal effects. The HSLS:09 has been described as the ideal dataset for examining determinants of student outcomes, such as who majors in STEM fields (Cimpian et al., 2020).

The survey followed a nationally representative sample of fall-term 9th-grade students in 2009 in more than 900 public and private high schools to 2012 (first follow-up) when most sample members were in 11th grade, and into higher education or the workplace (second follow-up). Students completed an in-person mathematics assessment focused on algebraic reasoning, as well as a survey that included items on educational experiences, sociodemographic background, educational expectations, mathematics and science student self-beliefs and interests, among other items. Students’ parents, principals, teachers, and school counselors participated in the surveys. In the first follow-up, which took place in the spring of 2012, each module from the base year was fielded again, except for the teacher questionnaires (U. S. Department of Education, 2018). The student-level files contain student responses and associated composite variables from the student, parent, and school administrator survey instruments. The school-level variables contain responses and associated derived variables from the school administrator and counselor instruments. In the public-use datafile, school and counselor/administrator survey data has already been merged into the student-level file.

The data files contain school and student level weights, including longitudinal weights appropriate for analyses which use variables from both the base year and the first follow-up (as is the case in this study) when conducting survey data analysis.

### Methodological approach

To estimate bidirectional effects between mathematics self-efficacy beliefs and mathematics achievement, two models were estimated (Model 1 and Model 2) separately by gender. In Model 1, earlier math self-efficacy is a potentially endogenous predictor of later math achievement. In Model 2, earlier math achievement is a potentially endogenous predictor of later math self-efficacy.

To address complications posed by endogenous covariates, we need to model potentially endogenous covariates, along with the outcome equation. This requires using one or more variables (“instruments”) that affect the endogenous covariate but can be excluded from the outcome equation. In estimating Models 1 and 2, I used instrumental variables (IV) estimation. Section “Model specification: Outcomes and covariates” discusses the excluded instruments used in each equation and testing of exclusion restrictions.

## Measures and model specification

Section “Measures” provides information on the derivation of measures which are central to the investigation, i.e., the self-efficacy composite scales, and the mathematics assessment scores. Section “Model specification: Outcomes and covariates” describes the covariates used in model estimation. It also discusses the excluded instruments used in the IV regressions, and how the relevance and validity of these instruments was tested.

### Measures

The self-efficacy composites in HSLS:09, given at two points in time (at grade 9 and 2.5 years later, at grade 11), are composite scales of the sample member’s perceived mathematics self-efficacy in a continuum of values from negative to positive, with higher values representing higher self-efficacy. They were generated by HSLS:09 project scientists through weighted principal component factor analysis and standardized to a mean of 0 and standard deviation of 1. The inputs to this scale were survey questions on how confident the student was in doing an excellent job on fall 2009 math tests, how certain the student was that he/she can understand math textbook, how certain the student was that he/she can master skills in fall 2009 math course, and how confident the student was on doing an excellent job on fall 2009 math assignments. There were four possible answers to these questions ranging from “Strongly Disagree” to “Strongly Agree.” The reported reliability (Cronbach’s α) of the math self-efficacy variable was 0.90 in the first wave and 0.89 in the second wave, which indicate a high level of internal consistency of the self-efficacy composite.

The mathematics assessment variables in the HSLS:09, provide a measure of student achievement in algebraic content domains and reasoning, upon entry to high school in fall 2009 and 2.5 years later. The scores used to assess students’ performance were based on Item Response Theory (IRT). The IRT model used patterns of correct, incorrect, and omitted responses to obtain ability estimates that are comparable across the low-, moderate-, and high-difficulty test forms (see, Ingels and Dalton, 2013, pp. A9-A11). This IRT estimate was then transformed to derive a final score (standardized *theta* score), the standard unit of the item response theory (IRT) model that represents the level of the domain being measured. The final achievement *theta* scores at grades 9 and 11 provide a summary measure of student mathematics achievement. The *theta* (ability) estimate at each point in time provides a summary measure of achievement. Scores from HSLS:09 first follow-up can be equated to the scale of HSLS:09 base year so that scores may be compared longitudinally. This is the case due to the common items between the HSLS:09 base year and first follow-up. The tests were equated using a procedure^3^ which allowed the base-year *thetas* to remain unchanged while the first follow-up *thetas* were equated to the existing base-year scale (U. S. Department of Education, 2018).

Across countries, boys generally (but not always) tend to outperform girls in mathematics tests, although differences are small; on the other hand, girls report lower mathematics self-efficacy than boys (e.g., Louis and Mistele, 2012). Table 1 presents summary statistics from the HSLS:09 for mean student math achievement, and math self-efficacy by grade and gender. In both the base year and the first follow-up, there were no statistically significant gender differences in mathematics achievement (*theta* ability score). On the other hand, statistically significant and non-trivial differences in perceived mathematics self-efficacy in favor of boys were observed in both years. Therefore, based on the raw data, while boys reported higher mathematics self-efficacy, boys and girls performed equally well.

**TABLE 1:** Self-efficacy and mathematics achievement by gender and grade.

Means	Grade 9			Grade 11		
		
	Male	Female	Δ (Male-Female)	Male	Female	Δ (Male-Female)
Math t*heta* score	0.092 (0.966)	0.087 (0.885)	0.005	0.685 (1.16)	0.657 (1.07)	0.027
Math self-efficacy composite	0.131 (1.00)	-0.054 (0.999)	**0.185**	0.119 (1.00)	-0.073 (0.994)	**0.192**

Standard deviations in parentheses; Bold indicates that differences significant at the 1% level or lower.

### Model specification: Outcomes and covariates

The mathematics (*theta*) score and the math self-efficacy composite are standardized (mean = 0, SD = 1). Model 1, in which Grade 11 math *theta* score is the outcome, includes the following categories of determinants of achievement: (1) Student demographic characteristics (age; location; minority race; immigrant status); (2) Student’s family characteristics (socioeconomic status; mother/father in a STEM occupation) (3) School characteristics (school ownership; school problems as reported by principal, as a proxy of school quality); (4) Teacher characteristics (math teacher’s sex; math teacher’s experience in teaching math); (5) Student’s interest in mathematics composite index; and (6) Student’s Grade 9 math-specific self-efficacy composite index, as a potentially endogenous determined covariate. I also control for early math ability using student’s earlier math performance (grade 9 math score) as proxy. It is important to properly control for earlier performance; while the survey contains an earlier measure of math performance, student’s grade 8 performance in the most advanced math course enrolled, performance is based on the course grade, not on a standardized test.

In the equation endogenously determining math self-efficacy, the excluded instruments are dummy variables which relate to students’ general beliefs on which gender is better in mathematics. These are: (1) “boys are better in math” and (2) “girls are better in math,” with “girls and boys are equally good in math” as the reference category. Such beliefs correlated with students’ assessment of their own efficacy in math. Specifically, believing that males are better in math are positively correlated with math self-efficacy for boys and negatively correlated for girls, while the opposite correlation pattern applied when believing that girls are better in math. Potential determinants of such student beliefs were investigated, by examining their relationship with various student background characteristics, such as parental beliefs on which gender is better in mathematics (based on related survey questions), parents’ education, and gender of math teacher. Statistically significant positive correlations were found between parents’ and students’ beliefs that males are better in math. Similarly, having a male math teacher is positively correlated with both male and female students’ belief that males are better in math. Furthermore, higher parental education (especially father’s education) is positively correlated with students believing that males are better in math.

Exclusion of these instruments from the outcome equation requires that, while such general beliefs correlate with students’ individual self-efficacy perceptions, they are not significant determinants of the outcome. From IV estimation of the Model 1, I assessed the relevance and exclusion restrictions associated with the excluded instruments using tests based on Sargan’s J-statistic. In addition, I used the recently developed *kinky least-squares* (KLS) approach by Jan Kiviet,^4^ which yields statistical inference on the validity of exclusion restrictions regarding candidate external instrument/s (single, or as a set) when a plausible range of endogeneity correlations is available, whereas these unavoidable restrictions were always supposed to be non-testable (Kiviet, 2020; Kripfganz and Kiviet, 2021).

Model 2 includes an outcome equation with the 11th grade self-efficacy composite as the outcome and the same controls as in the endogenous self-efficacy equation of Model 1. The equation modeling the potentially endogenous earlier mathematics achievement (mathematics *theta* score reported at grade 9), contains the following fixed characteristics as instruments: age of 9-grader (born in 1995 or later, vs. born in 1994); student/school location (town/village vs. larger location). The validity of the instruments requires that birth year and student/school location correlates math achievement but not with math-self-efficacy. I assessed the relevance and exclusion restrictions associated with the excluded instruments as in Model 1.

## Estimation and results

Sections “Model 1: The effect of prior math self-efficacy on later math achievement” and “Model 2: The effect of earlier achievement on later self-efficacy” present the estimation results for the effect of prior math self-efficacy on later math achievement (Section “Model 1: The effect of prior math self-efficacy on later math achievement”) and for the effect of earlier achievement on later self-efficacy (Section “Model 2: The effect of earlier achievement on later self-efficacy”). Section “Including teacher variables: comparison of estimates” compares estimates with and without inclusion of teacher variables in the vector of covariates.

### Model 1: The effect of prior math self-efficacy on later math achievement

Models were estimated with and without teacher variables (teacher sex and teacher years of experience teaching math) in the vector of exogenous covariates. Teacher variables contain a substantially higher proportion of missing values compared to the student information variables, resulting in about 25% more attrition; furthermore, it is not possible to impute missing values for teacher sex and experience. For example, the sample sizes in estimating Model 1 (6,813 and 7,045 for males and females) would have been 5,229 and 5,417, respectively. I, therefore, present the estimation results from models without teacher variables.^5^ A comparison of findings without and with teacher variables is given in Section “Including teacher variables: comparison of estimates.”

Table 2 contains the coefficient estimates for Model 1 by gender. The first column contains the Ordinary Least Squares (OLS) estimates (naïve model), with math self-efficacy an exogenous covariate. The second column reports the corresponding IV estimates, with a math self-efficacy the endogenous covariate.

**TABLE 2:** Effect of earlier mathematics self-efficacy on later mathematics achievement by gender.

	Male		Female	
		
Outcome: Grade 11 Math achievement (stand.)	OLS	IV	OLS	IV
Grade 9 math self-efficacy (stand.)	**0.089** (0.020)	**0.185** (0.087)	**0.080** (0.019)	0.095 (0.093)
Grade 9 math interest (stand.)	0.026 (0.015)	-0.018 (0.070)	0.014 (0.019)	-0.022 (0.080)
Born in 1995 or later	**0.092** (0.026)	**0.091** (0.027)	**0.082** (0.029)	**0.082** (0.029)
Town school	-0.054 (0.042)	-0.052 (0.042)	0.037 (0.038)	0.037 (0.038)
Village school	-**0.060** (0.029)	-**0.059** (0.029)	-0.032 (0.027)	-0.031 (0.027)
Black	-**0.138** (0.043)	-**0.152** (0.052)	-**0.100** (0.048)	-0.105 (0.072)
Hispanic	-**0.100** (0.046)	-**0.104** (0.044)	-0.077 (0.044)	-0.077 (0.045)
Asian	0.105 (0.059)	0.109 (0.059)	**0.264** (0.063)	**0.265** (0.065)
Another race	0.030 (0.180)	0.012 (0.186)	-0.064 (0.111)	-0.063 (0.111)
Born outside the US	**0.263** (0.067)	**0.260** (0.071)	-0.021 (0.084)	-0.021 (0.085)
Socioeconomic status index (stand.)	**0.105** (0.017)	**0.102** (0.017)	**0.098** (0.017)	**0.097** (0.017)
Father in STEM	**0.118** (0.049)	0.103 (0.055)	0.024 (0.049)	0.024 (0.049)
Mother in STEM	0.020 (0.054)	0.029 (0.056)	0.111 (0.061)	0.111 (0.062)
Public school	-**0.081** (0.033)	-**0.087** (0.034)	-**0.092** (0.032)	-**0.092** (0.033)
School problems index (stand.)	-**0.051** (0.014)	-**0.051** (0.014)	-**0.054** (0.016)	-**0.054** (0.016)
Grade 9 Math achievement (stand.)	**0.673** (0.016)	**0.653** (0.032)	**0.670** (0.017)	**0.667** (0.038)
Constant	-**0.092** (0.037)	-**0.091** (0.037)	-**0.112** (0.037)	-**0.110** (0.043)
*F*-value	251.6	250.7	209.4	207.8
**[***p*-value**]**	[0.000]	[0.000]	[0.000]	[0.000]

**First stage**:				

Females better in math	–	-**0.167** (0.045)	–	**0.133** (0.043)
Males better in math	–	**0.129** (0.036)	–	-**0.154** (0.043)
F test of excluded instruments:				
F-value	–	44.62	–	38.2
**[***p*-value**]**		[0.000]		[0.000]
Overidentification test				
Sargan’s J-statistic	–	0.46	–	0.01
**[***p*-value**]**		[0.498]		[0.920]
Test of endogeneity of Grade 9 math self-efficacy (H_0_: Grade 9 math self-efficacy exogenous)				
Wu-Hausman F-statistic	–	1.24	–	0.028
**[***p*-value**]**		[0.265]		[0.868]
*N*	6,813	6,813	7,045	7,045

Standard errors in parentheses. Bold indicates significance at the 5% level or lower.

#### Findings

In reporting the findings, the effect size metric, Beta (β), is the standardized regression coefficient, expressing the amount of expected change in the standardized outcome variable, associated with one standard deviation change in the predictor variable of interest.

The Ordinary Least Squares (OLS) estimate of the effect of higher earlier self-efficacy on later mathematics achievement is small; for males, it is estimated at nearly 0.1 SD higher Grade 11 math achievement for one SD increase in Grade 9 self-efficacy (β = 0.089, 95% CI: 0.05, 0.13), and slightly smaller for females (β = 0.08; 95% CI: 0.04, 0.11); the difference in point estimates by gender is not significant based on 95% confidence intervals. From these estimates, higher earlier mathematics self-efficacy is associated with higher later achievement, but the effects are small, approaching Cohen (1988) threshold of small effect size in social sciences. This is a general finding in the related empirical literature which is based on methodologies not suitable to derive estimates of causal effects.

Looking at the effect of other covariates with math achievement, earlier math interest does not display any association with late achievement. Significant associations are found for fixed characteristics such as race, socioeconomic status, parents’ occupation, school quality, along with earlier math achievement as a proxy of math ability. Black and Hispanic male students performed worse than White students (β = -0.14; *p*-value = 0.002 for Black and β = -0.1; *p*-value = 0.03 for Hispanic students), while Asian females performed better (β = 0.26; *p*-value = 0.000) and to a lesser extent Asian males (β = 0.1; *p*-value = 0.075). Male students born outside the US outperformed native students (β = 0.26; *p*-value = 0.000) but interestingly, there is no statistically significant difference by immigrant status among females. The positive effect of better socioeconomic status is small (β = 0.1; *p*-value = 0.000), while having a father in a STEM occupation is associated with higher achievement for boys (β = 0.12; *p*-value = 0.016), while having a mother in a STEM occupation is associated with higher achievement for girls (β = 0.11; *p*-value = 0.07). Finally, there is a strong association between earlier (Grade 9) and later (Grade 11) math achievement for both genders (β = 0.67 *p*-value = 0.000).

**CHART 1:**
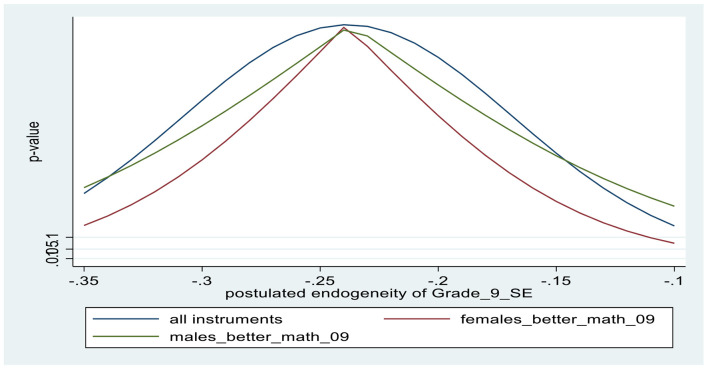
Tests of exclusion restrictions of instruments for Grade 9 math self-efficacy: MALES.

**CHART 2:**
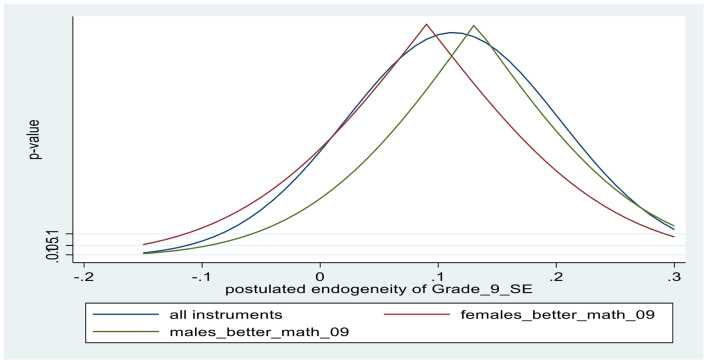
Tests of exclusion restrictions of instruments for Grade 9 math self-efficacy: FEMALES.

The lower panel of Table 2 contains the first-stage results from the IV regressions. The coefficient estimates of the two binary instruments show that, boys believing that boys (girls) are better in math correlates positively (negatively) with boys’ individual self-efficacy perceptions; similarly, girls believing that girls (boys) are better in math correlates positively (negatively) with girls’ individual self-efficacy perceptions. Based on the F-values in the first-stage test of excluded instruments (F = 44.6 in the male regression and 38.2 in the female regression), the instrument set is not weak. The overidentifying restrictions test (J-test) provides evidence that the excluded instruments are exogenous with high associated *p*-values. Charts 1, 2 provide additional evidence for the excludability of the instruments, using the *kinky least-squares* (KLS) approach (see Section “Model specification: Outcomes and covariates”). After deriving plausible ranges of endogeneity correlations (based on error correlation coefficients between the outcome and the self-efficacy equations), the charts depict the associated *p*-values for the validity of the exclusion restriction for each instrument alone, as well as the combination of instruments for various values of postulated endogeneity correlations. Finally, tests the endogeneity of math self-efficacy (Wu-Hausman F-test) suggest that the null hypothesis that math self-efficacy is exogenous is clearly accepted for females, while accepted with associated *p*-value = 0.26 for males.

Turning to the findings from the IV regressions (upper panel of Table 2), the point estimate for the effect of grade 9 math self-efficacy on Grade 11 math achievement for males is twice that from the OLS regression (β = 0.185; *p*-value = 0.034; 95% CI: 0.014, 0.36). However, the corresponding IV-point estimate for females is smaller (β = 0.095; *p*-value = 0.54; 95% CI: -0.21, 0.40), less precisely estimated, and of similar size to the OLS estimate. Due to the higher standards errors associated with IV estimates compared to those from the OLS estimates, the difference in IV estimates by gender is not statistically significant based on 95% confidence intervals. However, if the comparison is between the male IV estimate and the female OLS estimate (given that the null hypothesis of exogeneity of self-efficacy is accepted at high *p*-values for females), a difference in estimates by gender is established. From these findings, there is evidence in support of the *self-enhancement model* for males, while such evidence on females is weak.

### Model 2: The effect of earlier achievement on later self-efficacy

Table 3 contains the coefficient estimates for Model 2 by gender. From the OLS regression, the point estimate of Grade 9 math achievement on Grade 11 math self-efficacy (while controlling for Grade 9 math self-efficacy) is modest, but precisely estimated and of similar magnitude for males (β = 0.194; *p*-value = 0.000) and females (β = 0.21; *p*-value = 0.000).

**TABLE 3:** Effect of earlier mathematics achievement on later mathematics self-efficacy by gender.

	Males		Females	
		
Outcome: Grade 11 Math self-efficacy (stand.)	OLS	IV	OLS	IV
Grade 9 math achievement (stand.)	**0.194** (0.018)	**0.316** (0.122)	**0.207** (0.022)	0.164 (0.176)
Grade 9 math interest (stand.)	**0.099** (0.020)	**0.090** (0.023)	**0.058** (0.023)	**0.059** (0.025)
Black	**0.217** (0.051)	**0.269** (0.075)	**0.248** (0.064)	**0.233** (0.090)
Hispanic	0.041 (0.052)	0.059 (0.056)	0.047 (0.060)	0.042 (0.065)
Asian	-0.061 (0.076)	-0.115 (0.091)	-0.054 (0.084)	-0.029 (0.129)
Another race	-0.006 (0.188)	0.014 (0.202)	0.122 (0.204)	0.118 (0.206)
Born outside the US	-0.008 (0.078)	-0.046 (0.094)	0.094 (0.075)	0.092 (0.076)
Socioeconomic status index (stand.)	0.013 (0.020)	-0.022 (0.040)	-0.014 (0.020)	0.001 (0.054)
Females better in math	-0.052 (0.046)	-0.028 (0.052)	-0.045 (0.048)	-0.048 (0.050)
Males better in math	**0.117** (0.038)	**0.115** (0.040)	-0.081 (0.046)	-0.079 (0.047)
School problems index (stand.)	0.004 (0.016)	0.019 (0.021)	-0.017 (0.020)	-0.022 (0.029)
Grade 9 Math self-efficacy (stand.)	**0.232** (0.022)	**0.204** (0.038)	**0.227** (0.023)	**0.236** (0.045)
Constant	0.032 (0.022)	0.033 (0.022)	-**0.128** (0.023)	-**0.129** (0.024)
*F*-value	56.2	47.4	36.0	28.0
**[***p*-value**]**	[0.000]	[0.000]	[0.000]	[0.000]

**First stage**				

Born in 1995 or later	–	**0.183** (0.032)	–	**0.209** (0.035)
Town school	–	-**0.206** (0.050)	–	-
Village school	–	-**0.143** (0.032)	–	-**0.061** (0.032)
F test of excluded instruments:				
*F*-value		46.8		39.6
**[***p*-value**]**		[0.000]		[0.000]
Overidentification test: H_0_				
Sargan’s J-statistic	-	1.77	-	0.45
**[***p*-value**]**		[0.412]		[0.500]
Test of endogeneity of Grade 9 math achievement (H_0_: Grade 9 math achievement exogenous)				
Wu-Hausman F-statistic		1.67		1.36
**[***p*-value**]**		[0.196]		[0.243]
*N*	7,031	7,031	7,260	7,260

Standard errors in parentheses. Bold indicates significance at the 5% level or lower.

Before adjusting for math performance and other characteristics (i.e., based on summary statistics), Black and Asian students reported higher self-efficacy than White students. After adjusting for earlier math performance and other covariates, the Black-White difference in reported math self-efficacy increases further for both males (β = 0.22; *p*-value = 0.000) and females (β = 0.25; *p*-value = 0.000). On the other hand, after conditioning for covariates there is not statistically significant difference in reported self-efficacy between Asian and White students. In other words, Black students’ perceived efficacy in mathematics is higher than what would be consistent with their past math performance, while White and Asian students’ perceived efficacy is more in line with past performance. These findings are consistent with those in Bachman et al. (2011) on race/ethnic differences in self-esteem. They used nationally representative data of 8th- 10th- and 12th-grade students in the US, to find that African American students score highest, and Asian Americans score lowest; controlling for grades and college plans, heighten these race/ethnic differences. Furthermore, the findings are highly consistent over time. It is difficult to reconcile these race/ethnic differences in self-esteem using potential explanations based on social comparison processes (see Gray-Little and Hafdahl, 2000), or the reflected appraisals theory (see Crocker and Major, 1989), which would predict that ethnic minorities (such as African Americans) will have lower self-esteem than the ethnic majority. A more promising potential explanation relates to differences in cultural traditions and associated patterns of behavior. For example, Cai et al. (2007) suggest East Asian individuals, while feeling positively about themselves, cultural norms (such as modesty) account for observed differences in self-esteem scores. On the other hand, it has been suggested that African American families strive to instill self-esteem in youth, as a defense mechanism in coping with discrimination (e.g., Hughes et al., 2006; Bachman et al., 2011).

**CHART 3:**
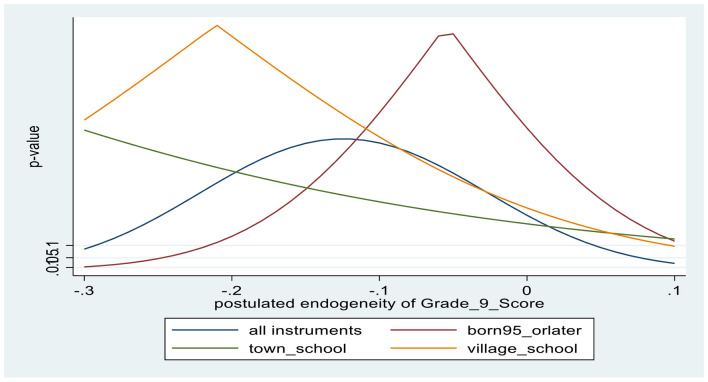
Tests of exclusion restrictions of instruments for Grade 9 math score: MALES.

**CHART 4:**
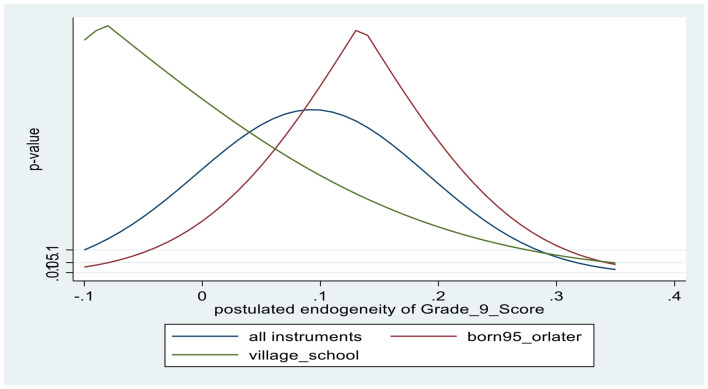
Tests of exclusion restrictions of instruments for Grade 9 math score: FEMALES.

Turning to the findings from IV regressions, from the first-stage results, slightly younger students (born in 1995) are associated with about 0.2 SD higher math achievement, while going to school in smaller communities is associated with about 0.15–0.2 SD lower math achievement. Based on the F-value in the first-stage test excluded instruments, the instrument sets are not weak (*F* = 46.8 in the male regression and *F* = 39.6 in the female regression). From the overidentifying restrictions test (J-test), there is evidence that the excluded instruments are exogenous, with associated *p*-values in the 0.4–0.5 range. Charts 3, 4 provide corroborative evidence for the excludability of the instruments, using the *kinky least-squares* (KLS) approach. Finally, tests the endogeneity of math self-efficacy (Wu-Hausman F-test) suggest that the null hypothesis that math self-efficacy is exogenous is accepted for both males and females; however, the associated *p*-values are relatively low, at about 0.2.

In the male regression, the IV estimate of the effect of higher earlier math achievement on later math self-efficacy (β = 0.32; *p*-value = 0.01; 95% CI: 0.08, 0.55), is larger than the OLS point estimate (β = 0.19; 95% CI: 0.16, 0.23). This IV estimate exceeds the threshold for weak effect size. However, the corresponding IV estimate for females (β = 0.16; *p*-value = 0.35; 95% CI: -0.18, 0.51) is smaller, less precisely estimated compared to the male estimate, and similar in size to the OLS estimate for females (β = 0.21; 95% CI: 0.16, 0.25). Due to higher standard errors of the IV estimates, gender differences in effect estimates can be established only at 80% or lower confidence intervals; however, the findings are at least suggestive of a higher effect size for males.

In conclusion, after considering potential endogeneity biases associated with earlier math achievement, based on the moderate size of the effect of earlier math achievement on later math self-efficacy, the findings provide support for the *skills development* hypothesis for males, but the evidence for females is weak.

### Including teacher variables: Comparison of estimates

Since the reported results are based on models without teacher variables, the coefficient estimates of interest were compared between the models without and with inclusion of teacher variables. With the teacher variables included in the vector of exogenous covariates in the model, having a more experienced math teacher is associated with a small and marginally statistically significant positive effect on students’ math achievement. Having a male math teacher was negatively associated with girls’ math performance but the association was weak, while no association between math performance and teacher’s gender was detected for males. Finally, no association between math self-efficacy and the two teacher variables was detected.

The coefficient estimates of earlier math self-efficacy in Model 1 and earlier math performance in Model 2 from the IV regressions without and with teacher variables, at least qualitatively; however, the estimated effects for earlier self-efficacy on later achievement (Model 1) are somewhat smaller for both males and females and less precisely estimated. For males, the estimated effect (β = 0.09; *p*-value = 0.46) is of similar magnitude to the OLS estimate, while the corresponding effect estimate for females (β = -0.03; *p*-value = 0.85) suggests that earlier math self-efficacy is not predictive of later achievement. In Model 2, the estimates from models with and without inclusion of teacher variables allow for the same conclusions; for males, the estimated effect mirrors that from the model without teacher variables (β = 0.33; *p*-value = 0.01) and is statistically significant at the 1% level. For females, the estimated effect is small and statistically insignificant (β = .04; *p*-value = 0.78). Furthermore, from 95% confidence intervals, the estimated effect for males is statistically larger than the corresponding effect for females. Concluding, based on the findings from the models with teacher variables, there is evidence of reciprocal effects for males, with the dominant effect from earlier math achievement to later math self-efficacy, while reciprocal effects cannot be established for females.

### Discussion

When assessing RQ1, findings suggest that in Model 1, weak evidence of endogeneity of earlier self-efficacy was found, but only for males. Comparing OLS and IV estimates using confidence intervals, the IV estimate (β = 0.185) is larger than the OLS estimate (β = 0.089) at conventional levels of significance. For females, on the other hand, exogeneity of earlier self-efficacy is clearly accepted, with both estimates essentially identical, at less than 0.1 SD higher math achievement for one SD increase in earlier math self-efficacy. With respect to RQ2 (is self-efficacy both a cause of and an effect of academic achievement?), reciprocal effects were established for male students, with the dominant effect from earlier achievement to later self-efficacy. For females, evidence in support of reciprocal effects is weak, especially in relation to the *self-enhancement* hypothesis. Finally, when assessing gender differences in effects (RQ3), comparing the OLS confidence interval for females to the IV confidence interval for males allows for the conclusion that the effect of earlier math self-efficacy on later math achievement is higher for males. However, gender differences in the effect of earlier math achievement on later math self-efficacy could not be established.

Before comparing the findings to those from earlier studies in the area of self-efficacy and achievement in math, note that this study differs from earlier studies in that it aims to derive estimates of causal effects by using both: (a) longitudinal data (as in a minority of related studies), from a large high-quality US dataset and multiple measurements at a 2.5-year interval and (b) Instrumental Variables (IV) methodology, which deals with potential endogeneity biases. This approach is better suited to derive causal effect estimates and identify gender differences in effects.

Earlier findings, such as those from the meta-analysis of longitudinal studies by Valentine et al. (2004), point to effect estimates which are consistent with a small favorable influence of positive self-beliefs on academic achievement (at about β = 0.1); this effect size meets or slightly exceeds Cohen’s definition of small effect size. These studies used various measures of self-beliefs, i.e., self-concept, self-efficacy, and self-esteem (but generally not math-specific), different measurement delay, number of control variables, matching vs. non-matching domains, and estimation method (such as multivariate regression, path analysis, and structural equation modeling). No significant gender differences were identified in the metanalysis, despite theoretical considerations relating to academic and non-academic concerns suggesting gender as a possible moderator. In this study I found that a stronger effect of earlier self-efficacy on later achievement for males compared to females, while the point IV estimate from the baseline model (without teacher variables) for males is larger than the OLS estimate, far exceeding the benchmark for small effect size.

Comparing findings on presence of reciprocal effects and effect dominance to past evidence, the findings bear some similarity to those by Hwang et al. (2016), who found a reciprocal relationship, with the effect of past academic achievement on later self-efficacy beliefs being stronger than the effect of self-efficacy beliefs on academic achievement. However, no gender differences were established in these studies. I provide evidence of reciprocal effects for male students with the dominant effect from earlier math academic achievement on later math self-efficacy; evidence of reciprocal effects for female students is weaker, with the dominant effect from earlier math academic achievement on later math self-efficacy as well.

The main findings of this study relate to differences by estimation method (generally higher effect estimates from IV compared to using OLS estimation) and related heterogeneity of effects by gender (larger effect estimates for males compared to females). The IV estimator accounts for confounding due to unobserved attributes and measurement error, which are not accounted for by the OLS estimator. One possible contributor to the heterogeneity of effects by gender is misreporting error in perceived math self-efficacy, since self-efficacy may be subject to systematic self-report bias. Specifically, there may be gender differences in misreporting/accurate reporting of math self-efficacy. While the standard case of measurement error pertains to problems with the “measurement tool,” here by systematic self-reporting bias I refer to non-random deviations between the self-reported and “true” values of the same measure (e.g., Bauhoff, 2011). The cognitive bias associated with over-estimating one’s ability, is known as the Dunning–Kruger effect (Kruger and Dunning, 1999). If “true” self-efficacy values are those consistent with students’ past mathematics achievements, reported self-efficacy values can be over/under-estimates of the “true” value. Using the standard non-classical “measurement error” literature,^6^ which occurs when the error in reporting the covariate of interest is correlated with the true value of that variable or with the errors in measuring those values (e.g., Bound et al., 1994), biases in any direction may arise (see for example, O’Neil and Sweetman (2012).

There are practical implications for the classroom from the findings in this study. Girls report lower mathematics self-efficacy than boys, while actual performance in mathematics does not differ significantly between genders. If the findings on gender differences in effects is associated with boys overestimating their past mathematics performance (positivity bias/positive illusions),^7^ while girls underestimate them or rate them more accurately, the result could be girls not pursuing math intensive courses. Increasing girls’ positivity bias in mathematics through feedback from teachers on their individual or group good past math performances toward bringing their perceptions into line with past achievements, is a promising intervention. Success of such an intervention requires teachers’ feedback, boosting future strengthening of self-efficacy perceptions of girls. This can boost girls’ willingness to pursue more advanced mathematics courses.

#### Limitations

The findings in this study can be generalized in the context of the relationship between mathematics-specific (academic) self-efficacy and mathematics performance in high school. One possible limitation to generalizing the findings, is that in the HSLS survey the mathematics domain on which assessment is based is somewhat narrowly defined (i.e., algebraic content domains and reasoning) so, one might hypothesize that with a different assessment domain, findings might have been somewhat different. Another limitation is that the derived IV estimates come with higher standard errors (as is usually the case); as a result, some of the differences in IV estimates (i.e., male vs. female) are suggestive, since differences cannot be demonstrated based on statistical significance at conventional levels of significance (for example, the difference in IV estimates by gender for the effect of earlier math self-efficacy on later math performance).

## Conclusion

This study aimed to first, investigate potential endogeneities in the relationship between students’ self-efficacy perceptions and their achievement in the domain of mathematics; second, after accounting for potential biases due to endogeneity, establish bidirectional causal relationships between self-efficacy and achievement; and third, identify differences by gender in effect estimates. Toward these aims, longitudinal data from the HSLS09 and first follow-up surveys on US high school students was used, along with Instruments Variables (IV) estimation. To uncover potential gender differences in effect estimates, models were estimated separately by gender.

The findings can be summarized as follows: (a) evidence for endogeneity of earlier self-efficacy as a predictor of later mathematics achievement and of earlier mathematics achievement as a predictor of later self-efficacy was found, but only for male students; (b) robust reciprocal effects from IV regressions were established only for male students, with the dominant effect from earlier achievement to later self-efficacy; and (c) while earlier correlational studies did not find significant gender differences in effects despite theoretical expectations for their existence, the findings in this study support higher effects for male students. Given that girls report lower mathematics self-efficacy than boys while their performance in mathematics does not differ significantly from that of boys, increasing girls’ positivity bias in mathematics through feedback from teachers on their individual/group good past math performances, is a promising intervention.

## Data availability statement

Publicly available datasets were analyzed in this study. This data can be found here: https://nces.ed.gov/surveys/hsls09/hsls09_data.asp.

## Author contributions

The author confirms being the sole contributor of this work and has approved it for publication.

## Footnotes

1 For example, unobserved mediators such as familial environment and peer influences may be involved when investigating earlier self-efficacy as a predictor of later achievement, while unobserved student innate academic ability or effort may be involved when investigating earlier achievement as a predictor of later self-efficacy.

2 While it is helpful to identify these “sources” separately, they are not truly distinct. For example, if a variable which truly measures self-efficacy is not available, this can be described as an omitted variables problem, or a problem associated with the available measure of self-efficacy being mismeasured/misreported. Understanding unobserved heterogeneity is facilitated by understanding endogeneity in a regression context.

3 The procedure used is the Stocking-Lord procedure (Stocking and Lord, 1983).

4 The main objective *kinky least-squares* estimation is to provide an instrument free alternative method of identifying coefficients and correcting ordinary least squares (OLS) biases, when a plausible range of postulated endogeneity correlation between a regressor and the error term (degree of endogeneity) is available.

5 The detailed results from models which include the two teacher variables are available upon request.

6 In the case of “classical” measurement error (error term as mean zero and is uncorrelated with the “true” dependent and the independent variables), the estimated coefficient from a naïve estimator, such as the OLS estimator) will be biased toward the null (attenuation bias).

7 This is suggested as a possible explanation, rather than having been demonstrated.
